# Sex hormone associations with breast cancer risk and the mediation of randomized trial postmenopausal hormone therapy effects

**DOI:** 10.1186/bcr3632

**Published:** 2014-03-26

**Authors:** Shanshan Zhao, Rowan T Chlebowski, Garnet L Anderson, Lewis H Kuller, JoAnn E Manson, Margery Gass, Ruth Patterson, Thomas E Rohan, Dorothy S Lane, Shirley AA Beresford, Sayeh Lavasani, Jacques E Rossouw, Ross L Prentice

**Affiliations:** 1 Public Health Sciences Division, Fred Hutchinson Cancer Research Center, 1100 Fairview Avenue North, P.O. Box 19024, Seattle WA, USA; 2 Los Angeles Biomedical Research Institute at Harbor-UCLA Medical Center, Torrance, CA, USA; 3 Department of Medicine, University of Pittsburgh, Pittsburgh, PA, USA; 4 Division of Preventive Medicine, Brigham and Women’s Hospital, Harvard Medical School, Boston, MA, USA; 5 University Hospital Menopause and Osteoporosis Center, University of Cincinnati, Cincinnati, OH, USA; 6 Department of Family and Preventive Medicine, University of California at San Diego, San Diego, CA, USA; 7 Department of Epidemiology and Population Health, Albert Einstein College of Medicine, Bronx, NY, USA; 8 Department of Preventive Medicine, State University of New York, Stony Brook, NY, USA; 9 Department of Epidemiology, University of Washington, Seattle, WA, USA; 10 Department of Oncology, Karmanos Cancer Center, Detroit, MI, USA; 11 National Heart, Lung and Blood Institute, National Institutes of Health, Bethesda, MD, USA

## Abstract

**Introduction:**

Paradoxically, a breast cancer risk reduction with conjugated equine estrogens (CEE) and a risk elevation with CEE plus medroxyprogesterone acetate (CEE + MPA) were observed in the Women’s Health Initiative (WHI) randomized controlled trials. The effects of hormone therapy on serum sex hormone levels, and on the association between baseline sex hormones and disease risk, may help explain these divergent breast cancer findings.

**Methods:**

Serum sex hormone concentrations were measured for 348 breast cancer cases in the CEE + MPA trial and for 235 cases in the CEE trial along with corresponding pair-matched controls, nested within the WHI trials of healthy postmenopausal women. Association and mediation analyses, to examine the extent to which sex hormone levels and changes can explain the breast cancer findings, were conducted using logistic regression.

**Results:**

Following CEE treatment, breast cancer risk was associated with higher concentrations of baseline serum estrogens, and with lower concentrations of sex hormone binding globulin. However, following CEE + MPA, there was no association of breast cancer risk with baseline sex hormone levels. The sex hormone changes from baseline to year 1 provided an explanation for much of the reduced breast cancer risk with CEE. Specifically, the treatment odds ratio (95% confidence interval) increased from 0.71 (0.43, 1.15) to 0.92 (0.41, 2.09) when the year 1 measures were included in the logistic regression analysis. In comparison, the CEE + MPA odds ratio was essentially unchanged when these year 1 measures were included.

**Conclusions:**

Breast cancer risk remains low following CEE use among women having favorable baseline sex hormone profiles, but CEE + MPA evidently produces a breast cancer risk for all women similar to that for women having an unfavorable baseline sex hormone profile. These patterns could reflect breast ductal epithelial cell stimulation by CEE + MPA that is substantially avoided with CEE, in conjunction with relatively more favorable effects of either regimen following a sustained period of estrogen deprivation. These findings may have implications for other hormone therapy formulations and routes of delivery.

**Trial registration:**

clinicaltrials.gov identifier: NCT00000611.

## Introduction

The Women’s Health Initiative (WHI) randomized, placebo-controlled postmenopausal hormone therapy (HT) trials were instrumental in leading to changes in clinical practice, including much reduced use of HT in the U.S. and around the world. Among the most important reasons for this change was the substantial elevation in breast cancer risk [1-4] among women assigned to active hormones in the CEE + MPA trial. In contrast, a modest reduction in breast cancer incidence [5-7] was observed among women assigned to active hormones in the CEE trial.

Considerable effort has been devoted to understanding these divergent breast cancer findings, through investigation of hazard ratio (HR) variations among trial cohort subsets [2,6] and through comparisons of HRs with those from the companion WHI Observational Study. The latter analyses reveal higher HRs among women who started HT soon after menopause [8,9], as has also been observed in other cohorts, for example, Beral *et al*. [10]. A recent report provides a detailed side-by-side presentation of CEE + MPA and CEE effects on breast cancer and other outcomes during the intervention and post-intervention phases of the WHI trials [11].

Serum sex hormone concentrations changed markedly following CEE or CEE + MPA administration in the WHI trial cohorts, with an approximate doubling of estradiol, estrone sulphate and sex hormone binding globulin (SHBG), and an approximate tripling of estrone with active treatment in each trial [12]. A recent report on a breast cancer nested case-control study within the CEE + MPA trial cohort noted that the treatment odds ratio was appreciably larger for women having relatively low baseline serum estrogens. For example, the OR (95% confidence interval (CI)) for breast cancer with CEE + MPA treatment was 2.47 (1.28, 4.79) among women in the lowest estradiol quartile, compared to 0.96 (0.44, 2.04) in the highest quartile [13].

Here we follow-up on this intriguing observation by examining the association between baseline serum sex hormones in the CEE + MPA and CEE trials and breast cancer risk, separately in the active treatment and placebo groups, during the intervention phases of the WHI trials. Concurrent associations of sex hormone changes from baseline to one year following randomization are also considered, and mediation analyses are conducted to examine the extent to which sex hormone changes can provide an explanation for the breast cancer findings in the two WHI trials.

## Methods

### Study population: breast cancer cases and controls

The design of the WHI Clinical Trial (CT) and corresponding baseline enrollee characteristics have been presented [14,15]. All women were postmenopausal and in the age range 50 to 79 when enrolled at 40 U.S. clinical centers during 1993 to 1998. The CT enrolled 68,132 women to either or both of a hormone therapy trial (27,347 women) or a low-fat dietary pattern trial (48,835 women). The CEE + MPA trial randomly assigned 16,608 women with uterus to 0.625 mg/d oral CEE plus continuous 2.5 mg/d MPA, or matching placebo. The CEE trial randomly assigned 10,739 women who were post-hysterectomy to this same oral estrogen preparation or placebo.

Women were excluded from the HT trials for breast cancer or other prior cancer (except non-melanoma skin cancer) within the past 10 years, baseline mammogram or clinical breast exam suggestive of cancer, or predicted survival of less than three years.

Women having ongoing or recent HT use at screening required a three-month washout period before becoming eligible.

The CEE + MPA trial intervention phase stopped early in July 2002, following an average of 5.6 years of intervention, when it was judged that health risks exceeded benefits. An elevation in breast cancer risk, in conjunction with an unfavorable global health risk versus benefit index, was key to the early stopping decision [1]. The CEE trial also stopped early in February 2004, primarily because of a stroke elevation of similar magnitude to that for CEE + MPA, following an average 7.1 years of intervention [5].

Clinical outcomes [16] were reported semi-annually in the CT through the end of the intervention period by self-administered questionnaires. Invasive breast cancer occurrences were confirmed by review of medical records and pathology reports by physician-adjudicators at local clinical centers. These events were classified centrally using NCI’s Surveillance, Epidemiology, and End Results coding system, including coding of histology, hormone receptor status and HER2 over-expression.

All women provided written informed consent for their various components of WHI participation, and for their participation in the postmenopausal hormone therapy randomized controlled trials. The protocol and informed consent documents and procedures were approved by the Institutional Review Board (IRB) of the Fred Hutchinson Cancer Research Center, and by the IRBs of each of the participating clinical centers. All research was conducted in compliance with the Helsinki Declaration.

There were 348 and 235 (invasive) breast cancer cases having sufficient serum for sex hormone analyses during the intervention phases of the CEE + MPA and CEE trials, respectively. Each was matched to a control from the same trial cohort who was without breast cancer during the trial intervention phase, on age at screening (within one year), race (white, black, Hispanic, other) and date of randomization (within 30 days).

### Blood samples and measurements of endogenous sex hormones

Blood samples were collected at baseline and year 1 after an overnight fast of at least 12 hours [17]. Specimens were separated and stored at -70°C within two hours of collection prior to shipping to a central repository. Baseline and one-year serum specimens from a case and matched control were batched together, and sent to the Reproductive Endocrine Research Laboratory (University of Southern California, Los Angeles, CA, USA) for sex hormone assessment. Laboratory personnel were blinded to case versus control status, and to baseline versus one-year collections.

Total estradiol and estrone concentrations were quantified by radioimmunoassays (RIA) using methods previously\described [13]. Estrone sulfate was measured by direct RIA using a commercial kit (Beckman Coulter, Minneapolis, MN, USA). SHBG was quantified by use of a direct chemiluminescent immunoassay using the Immulite Analyzer (Siemens Medical Solutions Diagnostics, Malvern, PA, USA). Bioavailable (non-SHBG-bound) estradiol concentration was calculated by an algorithm that uses the total estradiol and SHBG measurements [13].

Split sample quality control specimens, derived from women who were screened for WHI participation but did not enroll, were included in each assay batch. There were 60 such samples selected for the baseline hormone assays, and 60 for the year 1 assays in this case-control study. The quality control specimens revealed substantial variations between blind duplicate samples for a small fraction of pairs, particularly at low concentrations. However, the intra-assay coefficients of variation for the various assays were acceptable, and ranged from 4.2% to 13.4%, after excluding outliers that had differences between duplicates following log-transformation that exceeded twice the interquartile range (IQR). The assay sensitivities for estradiol, estrone, estrone sulfate and SHBG were 2, 4 and 50 pg/ml, and 1 Nmol/L, respectively.Study subjects were excluded from analyses for each analyte if one or both of the baseline and year 1 assessments did not yield a measurement. The number of individuals excluded in the CEE + MPA trial was 49 for estradiol, 61 for bioavailable estradiol, 49 for estrone, 105 for estrone sulfate and 46 for SHBG. The corresponding numbers for the CEE trial were 35, 43, 33, 85 and 36.

### Statistical methods

Serum hormone measurements were log-transformed to achieve approximate normal distributions. In response to the split sample blind duplicate data mentioned above, cases or controls were excluded for a specific analyte if the logarithm of the ratio of one-year to baseline values was outside of the 25^th^ to 75^th^ percentiles of the data by more than twice the IQR, separately by trial and randomization group.

OR modeling relied on logistic regression with case (1) or control (0) defining the binary ‘response’ variable [18]. Odds ratios were estimated as a function of (log-transformed) baseline and year 1 concentrations. For ease of interpretation, ORs corresponding to a doubling of the serum hormone concentrations are presented. Mediation was assessed by comparing the post-year 1 breast cancer OR associated with hormone therapy when only baseline serum hormones were included in the model, to the corresponding hormone therapy OR when year 1 serum hormone concentrations, or equivalently the ratio of year 1 to baseline concentrations, were added to the OR model. Mediation is indicated by hormone therapy ORs that move substantially toward the null when the year 1 analyte data are included. In addition to the matching factors of age and race, the logistic regression analyses included baseline body mass index (BMI), family history of breast cancer, cigarette smoking history and Gail model five-year breast cancer risk score [19] as control variables.

Nominal 95% CIs are presented for odds ratios, and all significance levels (*P*-values) are two-sided.

## Results

Table 1 presents distributional information for breast cancer cases and controls, separately for the CEE and CEE + MPA trials, in relation to several baseline characteristics. The average age for cases was about 64 years, and average BMI was about 30.

**Table 1:** Mean ± SE for continuous baseline covariates, and number (%) for categorical baseline covariates for breast cancer cases and controls occurring during the intervention phases of the Women’s Health Initiative CEE + MPA and CEE trials

	**CEE + MPA trial**			**CEE trial**		
	**Cases**	**Controls**	*P*-value*	**Cases**	**Controls**	*P*-value*
N	348	348		235	235	
Age^†^	64.32 ± 6.85	64.33 ± 6.82	0.98	64.5 ± 7.11	64.49 ± 7.06	0.97
BMI	29.33 ± 5.56	28.46 ± 5.69	0.04	31.46 ± 5.98	29.32 ± 5.70	<0.01
Gail model five-year risk score (%)	1.85 ± 0.89	1.72 ± 0.84	0.05	1.84 ± 1.65	1.36 ± 0.90	0.08
Race (%)^†^			0.99			0.98
White	305 (87.6)	305 (87.6)		184 (78.3)	184 (78.3)	
Black	21 (6.0)	21 (6.0)		36 (15.3)	35 (14.9)	
Other	22 (6.3)	22 (6.3)		15 (6.4)	16 (6.8)	
Smoking (%)			0.11			0.72
Never	158 (46.1)	186 (54.1)		129 (55.1)	126 (53.6)	
Past	155 (45.2)	131 (38.1)		87 (37.2)	86 (36.6)	
Current	30 (8.7)	27 (7.8)		18 (7.7)	23 (9.8)	
Family history of breast cancer (%)			0.006			0.23
Yes	79 (24.0)	50 (15.2)		52 (23.5)	42 (18.5)	
No	250 (76.0)	280 (84.8)		169 (76.5)	185 (81.5)	

*P-values are significance levels from Student’s *t*-test and chi-square tests of no difference between cases and controls for continuous and categorical variables.

^†^Matching variables in control selection. BMI, body mass index; CEE, conjugated equine estrogens; MPA, medroxyprogesterone acetate.

Subsequent analyses exclude 31 breast cancer cases in the CEE + MPA trial and 16 cases in the CEE trial that occurred during the first year from randomization. The log-transformed ratios of year 1 to baseline values had approximately normal distributions for each sex hormone, but with a small fraction of outlying values, as anticipated from the blind duplicate data. Applying the interquartile range criterion (see Methods) led to the further exclusion of the following number of placebo cases or controls in the CEE + MPA trial: estradiol - 20, bioavailable estradiol - 25, estrone - 25, estrone sulfate - 28, SHBG - 21. Corresponding numbers in the active treatment group in the CEE + MPA trial were 11, 19, 11, 7 and 10; with the smaller number of exclusions consistent with the notion that measurement reliability is primarily an issue at low concentrations. The corresponding numbers of cases or controls excluded from the CEE trial placebo group on the basis of this IQR criterion were 31, 30, 17, 16 and 31, and from the CEE trial active treatment group were 9, 10, 5, 10 and 3.

Tables 2 and 3 show geometric means and 95% confidence intervals for cases and controls for each analyte, separately for the placebo and active groups in the CEE + MPA trial and the CEE trial. In the placebo group in either trial one sees the expected positive association between baseline or year 1 serum estrogens, and the inverse association of baseline SHBG, with disease risk. These same patterns prevail in the active treatment group in the CEE trial. In contrast, these patterns are not at all evident in the active treatment group in the CEE + MPA trial, suggesting that the use of CEE + MPA overrides the expected associations of baseline sex hormone concentrations with breast cancer risk.

**Table 2:** Geometric mean and 95% confidence interval for sex hormone concentrations, separately for placebo and active randomization groups for breast cancer cases and controls from the Women’s Health Initiative CEE + MPA trial

	**CEE + MPA trial**					
	**Placebo**			**Active treatment**		
	**Cases**	**Controls**	*P*-value*	**Cases**	**Controls**	*P*-value*
Estradiol (pg/ml)						
Baseline	11.97 (4.96, 28.84)	10.05 (4.36, 23.17)	0.001	10.90 (4.83, 24.59)	10.89 (4.19, 28.34)	0.99
Year 1	10.12 (3.56, 28.71)	8.46 (2.86, 25.02)	0.008	22.30 (7.88, 63.06)	20.78 (6.02, 71.72)	0.28
Bioavailable estradiol (pg/ml)						
Baseline	8.02 (2.89, 22.25)	6.47 (2.49, 16.85)	0.001	7.10 (2.71, 18.60)	7.00 (2.31, 21.16)	0.82
Year 1	6.75 (2.16, 21.06)	5.35 (1.52, 18.76)	0.002	9.95 (3.75, 26.38)	9.98 (3.14, 31.71)	0.96
Estrone (pg/ml)						
Baseline	41.81 (17.36, 95.94)	35.20 (14.99, 82.68)	0.007	36.95 (16.06, 85.03)	36.37 (15.79, 83.74)	0.74
Year 1	37.68 (13.99, 101.49)	34.39 (13.78, 85.81)	0.14	117.48 (30.97, 445.66)	103.96 (23.00, 469.86)	0.14
Estrone sulfate (ng/ml) baseline	0.84	0.79	0.26	0.82	0.80	0.65
	(0.37, 1.91)	(0.33, 1.88)		(0.33, 2.08)	(0.29, 2.18)	
Year 1	0.75 (0.34, 1.67)	0.69 (0.30, 1.59)	0.17	1.88 (0.61, 5.81)	1.77 (0.51, 6.14)	0.37
SHBG (Nmol/L)						
Baseline	36.93 (15.05, 90.63)	41.94 (17.28, 101.80)	0.02	40.49 (14.99, 109.42)	39.81 (14.63, 108.31)	0.76
Year 1	37.37 (14.22, 98.23)	41.86 (16.51, 106.11)	0.06	95.46 (31.96, 285.11)	90.07 (30.96, 262.06)	0.35

Samples are excluded if changes from baseline to year 1 log-transformed concentrations are outside the interquartile range by twice its width.

*P-value from Student’s *t*-test comparison of case versus control values. CEE, conjugated equine estrogens; MPA, medroxyprogesterone acetate; SHBG, sex hormone binding globulin.

**Table 3:** Geometric mean and 95% confidence interval for sex hormone concentrations, separately for placebo and active randomization groups for breast cancer cases and controls from the Women’s Health Initiative CEE trial

	**CEE trial**					
	**Placebo**			**Active treatment**		
	**Cases**	**Controls**	*P*-value*	**Cases**	**Controls**	*P*-value*
Estradiol (pg/ml)						
Baseline	12.36 (5.59, 27.38)	10.56 (4.36, 25.57)	0.01	12.72 (3.88, 41.67)	10.43 (4.30, 25.32)	0.01
Year 1	10.31 (3.55, 29.94)	9.37 (3.64, 24.14)	0.21	26.47 (7.89, 88.76)	23.96 (6.77, 84.84)	0.27
Bioavailable estradiol (pg/ml)						
Baseline	8.53 (3.41, 21.33)	6.76 (2.59, 17.65)	0.001	8.74 (2.53, 30.20)	6.54 (2.27, 18.84)	0.001
Year 1	6.99 (2.29, 21.28)	6.05 (2.21, 16.57)	0.08	12.33 (3.66, 41.57)	10.21 (3.26, 32.02)	0.03
Estrone (pg/ml)						
Baseline	41.05 (17.54, 96.08)	36.23 (15.48, 84.79)	0.05	41.25 (16.66, 102.15)	36.44 (15.66, 84.80)	0.05
Year 1	38.54 (14.68, 101.17)	33.70 (12.06, 94.13)	0.08	124.39 (31.09, 497.59)	134.65 (30.72, 590.11)	0.44
Estrone sulfate (ng/ml)						
Baseline	0.86 (0.41, 1.81)	0.77 (0.33, 1.79)	0.07	0.80 (0.29, 2.20)	0.77 (0.35, 1.69)	0.59
Year 1	0.76 (0.51, 1.49)	0.62 (0.43, 1.49)	0.001	1.68 (0.65, 6.84)	1.70 (0.69, 7.06)	0.94
SHBG (Nmol/L)						
Baseline	34.11 (14.47, 80.39)	39.99 (15.30, 104.52)	0.02	34.05 (13.11, 88.43)	42.79 (15.19, 120.50)	0.002
Year 1	34.54 (15.30, 77.95)	40.86 (15.73, 106.12)	0.01	82.34 (24.57, 276.00)	99.61 (28.47, 348.54)	0.03

Samples are excluded if changes from baseline to year 1 log-transformed concentrations are outside the interquartile range by twice its width.

*P-value from Student’s *t*-test comparison of case versus control values. CEE, conjugated equine estrogens; SHBG, sex hormone binding globulin.

Table 4 presents a refined version of the baseline sex hormone associations with breast cancer in the two HT trials. Odds ratios for a two-fold increment in baseline sex hormone level are presented from logistic regression analyses that control for various breast cancer risk factors (see Methods), in separate analyses for each analyte. Positive associations with most baseline estrogens and an inverse association with SHBG are evident in the placebo groups and in the active treatment group in the CEE trial. However, these associations are not evident in the active treatment group in the CEE + MPA trial.

**Table 4:** Odds ratios (95% confidence intervals (CI)) for a doubling of baseline sex hormone levels in the randomized placebo and active treatment groups of the Women’s Health Initiative postmenopausal hormone therapy trials

	**Odds ratio (95% CI)**		**Numbers of cases/controls**
	**Placebo**	**Active**	
CEE + MPA trial*			
Estradiol	2.00 (1.24, 3.21)	0.96 (0.66, 1.40)	276/314
Bioavailable estradiol	1.98 (1.27, 3.07)	1.01 (0.72, 1.43)	265/299
Estrone	1.77 (1.15, 2.73)	1.07 (0.74, 1.56)	273/313
Estrone sulfate	1.18 (0.78, 1.80)	1.08 (0.78, 1.49)	245/287
SHBG*	0.75 (0.51, 1.12)	1.14 (0.82, 1.59)	274/323
CEE trial*			
Estradiol	1.50 (0.85, 2.65)	1.31 (0.88, 1.97)	172/207
Bioavailable estradiol	1.69 (0.99, 2.87)	1.58 (1.06, 2.36)	169/203
Estrone	1.28 (0.78, 2.10)	1.31 (0.83, 2.07)	186/216
Estrone sulfate	1.58 (0.91, 2.73)	1.05 (0.65, 1.70)	160/184
SHBG*	0.65 (0.39, 1.08)	0.61 (0.40, 0.92)	178/208

*CEE + MPA, conjugated equine estrogens plus medroxyprogesterone acetate; CEE, conjugated equine estrogens; SHBG, sex hormone binding globulin.

Table 5 examines the ability of sex hormone changes from baseline to year 1 following randomization to mediate the divergent effects of the two hormone therapy regimens on breast cancer incidence. Odds ratios for a two-fold increment in baseline sex hormones are presented, along with odds ratios for a two-fold increment (‘change’) from baseline to year 1. The upper part of Table 5 includes bioavailable estradiol and SHBG jointly, while the lower part includes the other four serum estrogen measures simultaneously along with SHBG (bioavailable estradiol is calculated from the other measures and can not be included with them). The two parts of the Table provide a similar message: inclusion of the baseline to year 1 sex hormone changes in the regression model has little impact on the treatment odds ratio for CEE + MPA, but doing so appreciably increases the treatment OR for CEE toward the null. For example, in the lower part of Table 5, inclusion of the baseline to year 1 change variables in the analysis moves the estimated CEE + MPA treatment OR slightly away from the null, from 1.59 to 1.65, whereas the estimated CEE treatment OR moves substantially toward the null, from 0.71 to 0.92. While the upper part of Table 5 suggests that the SHBG increase with CEE may compensate somewhat for corresponding serum estrogen increases, the more detailed analysis in the lower part of Table 5 suggests that women having relatively large increases in serum estrone with CEE are at reduced breast cancer risk.

**Table 5:** Odds ratios (95% confidence intervals (CI)) for hormone therapy treatment assignment, and for a doubling of baseline sex hormone and for a doubling from baseline to year 1 of sex hormone values in the Women’s Health Initiative postmenopausal hormone therapy trials

	**Odds ratio (95% confidence interval)**			
	CEE + MPA trial*		CEE trial*	
	**Baseline only†**	**Baseline + year 1†**	**Baseline only**	**Baseline + year 1**
Treatment	1.58 (1.13, 2.22)	1.46 (0.86, 2.47)	0.62 (0.40, 0.96)	0.84 (0.43, 1.65)
Baseline:				
Bioavailable estradiol	1.41 (1.03, 1.93)	1.44 (1.03, 2.01)	1.31 (0.93, 1.84)	1.24 (0.86, 1.79
SHBG*	1.11 (0.83, 1.50)	1.13 (0.83, 1.53)	0.67 (0.47, 0.97)	0.64 (0.44, 0.83)
Baseline to year 1 change:				
Bioavailable estradiol		1.05 (0.81, 1.36)		0.87 (0.62, 1.23)
SHBG		1.04 (0.76, 1.43)		0.85 (0.57, 1.26)
Cases/controls	254/291	254/291	161/191	161/191
	**CEE + MPA trial**		**CEE trial**	
	**Baseline only**	**Baseline + year 1**	**Baseline only**	**Baseline + year 1**
Treatment	1.59 (1.10, 2.30)	1.65 (0.92, 2.97)	0.71 (0.43, 1.15)	0.92 (0.41, 2.09)
Baseline:				
Estradiol	1.22 (0.71, 1.76)	1.22 (0.71, 2.08)	1.56 (0.90, 2.71)	1.87 (0.97, 3.62)
Estrone	1.28 (0.82, 2.01)	1.20 (0.72, 2.02)	0.78 (0.43, 1.40)	0.57 (0.29, 1.12)
Estrone sulfate	0.95 (0.69, 1.30)	0.93 (0.66, 1.31)	1.09 (0.69, 1.73)	1.25 (0.76, 2.05)
SHBG	0.92 (0.69, 1.23)	0.92 (0.67, 1.25)	0.56 (0.38, 0.83)	0.60 (0.39, 0.90)
Baseline to year 1 change:				
Estradiol		1.11 (0.79, 1.56)		1.18 (0.71, 1.96)
Estrone		0.95 (0.65, 1.39)		0.57 (0.35, 0.95)
Estrone sulfate		0.94 (0.65, 1.37)		1.50 (0.91, 2.48)
SHBG		1.00 (0.66, 1.50)		1.00 (0.56, 1.79)
Cases/controls	230/248	230/248	147/151	147/151

*CEE + MPA, conjugated equine estrogens plus medroxyprogesterone acetate; CEE, conjugated equine estrogens; SHBG, sex hormone binding globulin.

^†^For each trial the left column gives odds ratios and 95% confidence intervals when only baseline variables are included, while the right column adds corresponding year 1 variables in the analysis. Hormone therapy mediation is indicated by treatment odds ratios that move substantially toward the null when the year 1 sex hormone data are included.

## Discussion

These analyses provide insight into the divergent breast cancer findings with CEE + MPA versus CEE in the WHI randomized, controlled trials. Specifically, women at a relatively low baseline breast cancer risk in the CEE trial continue at low risk if assigned to active treatment. Furthermore, the configuration of sex hormone changes among women assigned to active CEE provides an explanation for much of the observed breast cancer risk reduction (Table 5).

In sharp contrast, there is no evidence for any association between baseline sex hormone levels and breast cancer risk among women assigned to active treatment in the CEE + MPA trial, whereas associations are apparent in the placebo group (Table 4). Moreover, breast cancer risk does not relate to changes in sex hormones from baseline to year 1 in the CEE + MPA trial (Table 5). Evidently, in the presence of 2.5 mg/d medroxyprogesterone acetate, these risk variations are quite unimportant. Women having a favorable serum sex hormone profile, perhaps from years of good diet and activity patterns and normal weight maintenance, evidently have similar elevated breast cancer risk to those having an unfavorable serum sex hormone profile. These observations provide an explanation for the previously reported interaction between baseline sex hormone levels and CEE + MPA odds ratios [13].

The analyses presented here excluded about 10 to 15% of placebo group, and about 5% of active treatment group cases and controls based on an IQR outlier criterion as applied to the differences between baseline and one-year log-analyte concentrations. These exclusion rates are consistent with a poor correspondence between split sample quality control specimens for about 10% of samples, especially at low sex hormone concentrations. To ensure that our outlier exclusion method was not unduly influencing results, the Tables 2, 3, 4 and 5 analyses were repeated using Rosner’s [20] ‘many-outlier detection procedure’, which relies on departure from normality. This method resulted in fewer outlier exclusions, about 2 to 4% in the placebo groups and almost none in the active treatment groups. The analytic results with this less restrictive outlier detection approach were quite similar to those shown in Tables 2, 3, 4 and 5. A modest difference arose in the Table 5 analysis where the HR (95% CI) for a doubling of estrone from baseline to one-year was 0.68 (0.44, 1.05), as compared to 0.57 (0.35, 0.95) in Table 5.

The mechanisms whereby MPA would have such a dominant effect on breast cancer risk remain to be elucidated. MPA is a potent progestin with high affinity for progesterone and androgen receptors, and with little estrogen receptor affinity. It circulates in a form that is 88% bound to albumin, and is not bound by SHBG [21]. MPA could exert direct intracellular effects on division rates of breast ductal epithelial cells in a manner that achieves a high cell division rate regardless of the other sex hormone concentrations. A related possibility is that MPA sensitizes the proliferative response to low doses of estradiol [13].

The analyses presented here suggest that the collective changes in sex hormones with CEE contribute strongly to the observed breast cancer risk reduction [5-7] in the WHI trial. It is plausible that the roughly two-fold increase in SHBG with CEE substantially offsets the corresponding large increase in serum estrogens in their effects on breast ductal epithelial cells. The upper portion of Table 5 provides some modest support for this line of reasoning, and may raise concerns about potential breast cancer risks with the trend toward use of transdermal estradiol, which does not materially increase SHBG in a first-pass hepatic effect, but substantially increases serum estradiol. Moreover, both oral and transdermal estradiols increase serum estradiol to a greater extent than does CEE.

The detailed analyses shown in the lower portion of Table 5 suggest that an additional mechanism may be at play with CEE. Specifically, women having a relatively large estrone increase with CEE use have a reduced (*P* = 0.03) breast cancer risk. This observation could align with the intriguing hypothesis that estrogen exposure following a sufficient period of estrogen deprivation induces apoptosis of nascent breast tumor cells [22]. Note, however, that the increases in SHBG and serum estrogens with CEE use are highly correlated (for example, correlations (95% CIs) of 0.73 (0.66, 0.79) between (log-transformed) estradiol and estrone changes; and 0.59 (0.59, 0.67) between estrone and SHBG changes in the control group women) making it challenging to distinguish among the changes in specific sex hormones in relation to breast cancer risk. The fact that the estrone change stands out in Table 5, in spite of these statistical challenges, is interesting and merits replication in other settings.

The strengths of this study are the randomized, controlled design of the WHI hormone therapy trials of adequate size, the quality sex hormone data assessment, and the ability to study CEE and CEE + MPA results side-by-side. Weaknesses include the absence of measurements on the biological changes resulting from the use of MPA.

## Conclusions

In summary, post-treatment changes in serum estrogens and SHBG concentrations, or changes in the association of such concentrations with disease risk, have the potential to substantially explain both the elevation in breast cancer risk with CEE + MPA and the reduction in risk with CEE. The CEE data suggest that a roughly two-fold increase in SHBG may offset risk that may otherwise attend corresponding major serum estrogen increases, and also support the observation [9] that estrogen exposure following a sustained period of estrogen deprivation reduces risk [22]. However, when MPA is added to the hormone therapy regimen, the risk variations otherwise associated with these sex hormone levels are no longer evident, and an important increase in breast cancer risk follows, especially among women who would otherwise be at relatively low risk. Whether different formulations or routes of delivery of HT will provide similar results is unknown and also warrants further study.

## Abbreviations

BMI: body mass index; CEE: conjugated equine estrogens; CEE + MPA: CEE plus medroxyprogesterone acetate; CI: confidence interval; CT: clinical trial; HR: hazard ratio; HT: hormone therapy; IQR: interquartile range; IRB: Institutional Review Board; OR: odds ratio; SHBG: sex hormone binding globulin; WHI: Women’s Health Initiative.

## Competing interests

The authors declare that they have no competing interests.

## Authors’ contributions

RC, GA, LK, JM, MG, RP (Patterson), DL, SB, JR and RLP (Prentice) were lead investigators from early in the WHI, and were active in the conception and design of the HT trials. These authors, as well as SZ, TR and SL, were actively involved in the analysis and interpretation of data for the present work. SZ and RLP developed an initial draft of the manuscript, and SZ was the principal data analyst. All authors (SZ, RC, GA, LK, JM, MG, RP, TR, DL, SB, SL, JR and RLP) participated in critical revisions leading to the present version. All authors read and approved the final version of the manuscript.
